# TRANCE is a unique marker of chronic pancreatitis in children

**DOI:** 10.1172/jci.insight.198911

**Published:** 2026-08-24

**Authors:** Peter R. Farrell, Bomi Lee, Faizan Ahmed, Maria E. Moreno-Fernandez, Ajay Dixit, Juan Pablo Gurria, Nicholas J. Ollberding, Qing Duan, Vineet Garlapally, Phoebe Christian, Sohail Z. Husain, Maisam Abu-El-Haija

**Affiliations:** 1 Division of Gastroenterology, Hepatology, and Nutrition, Cincinnati Children’s Hospital Medical Cincinnati, Ohio, USA.; 2 Department of Pediatrics, University of Cincinnati College of Medicine, Cincinnati, Ohio, USA.; 3 Department of Pediatrics, Division of Gastroenterology, Hepatology, and Nutrition, School of Medicine, Stanford University, Stanford, California, USA.; 4 Department of Surgery, University of Cincinnati College of Medicine, Cincinnati, Ohio, USA.; 5 Division of Pediatric General and Thoracic Surgery, and; 6 Division of Biostatistics and Epidemiology, Cincinnati Children’s Hospital Medical Center, Cincinnati, Ohio, USA.; 7 Stanford Medicine Children’s Health, Stanford, California, USA.

**Keywords:** Bone biology, Gastroenterology, Inflammation, Biomarkers, Bone development, Cytokines

## Abstract

**BACKGROUND:**

Elucidating immune signals through well-defined cohorts of pediatric acute pancreatitis (AP) and chronic pancreatitis (CP) patients is critical. This study aimed to evaluate plasma chemokine and cytokine levels in pediatric participants with CP, compared with AP and healthy controls (HCs), to identify unique biomarkers of CP.

**METHODS:**

Individuals were identified from a prospectively collected pediatric cohort (*n* = 146). Immunoproteins (*n* = 247) were measured using the NULISAseq platform on samples from individuals with CP (*n* = 71), AP (*n* = 55), and HCs (*n* = 20).

**RESULTS:**

The measured analytes showed separation among the 3 groups (*R*^2^ = 0.13, *P* < 0.001). In the CP group, TRANCE, TWEAK, FLT-1, HGF, and TRAIL were increased when compared with HC and AP patients (FDR-corrected *P* <0.05). A multivariable logistic regression model including all 5 proteins provided an AUC of 0.94 (0.93–0.96) for differentiating samples from CP versus AP or HC samples. In the AP group, CRP, IL-6, CD3E, ENRAGE, and MIF were elevated compared with HCs, while FGF-2, TAFA-5, IL-33, TRANCE, and CXCL12 were downregulated in the same acute time period (FDR-corrected *P* < 0.05). In the 21 patients with AP for whom follow-up samples were obtained, there was a notable decrease in sequential expression of IL-6 and CRP over 12 months and increased expression of CCL25, TAFA-5, and TRANCE proteins. Additionally, TRANCE was expressed on CP pancreatic tissue.

**CONCLUSIONS:**

TRANCE was increased in pediatric patients with CP and decreased in those with AP during a flare. Future studies are needed to investigate the role of TRANCE and other analytes in the pathogenesis of CP.

## Abstract 2

It can be hard to diagnose chronic pancreatitis in children. TRANCE is elevated in plasma samples from pediatric chronic pancreatitis compared to other participants and therefore may serve as a unique biomarker of the disease.

## Introduction

Chronic pancreatitis (CP) in children is a progressive fibroinflammatory disease characterized by irreversible morphological changes in the pancreatic tissue, leading to chronic abdominal pain and, in many cases, exocrine pancreatic insufficiency and diabetes (1, 2). This condition can start very early in life and affect children from young ages. While the progressive nature of the disease is similar to adult pancreatitis, the etiologies underlying it are often quite different, with pediatric CP mostly associated with genetic and anatomic/obstructive risk factors (2–4). It has been well described that patients with acute pancreatitis (AP) are at risk for progression to CP; however, early identification and management remain substantial clinical challenges in CP. The INSPPIRE (International Study Group of Pediatric Pancreatitis: In Search for a Cure) was established to study the risk factors and guide management of pediatric pancreatitis patients. Still, there remains limited data regarding the biochemical changes and blood biomarkers associated with CP in pediatrics (1). The current definition of CP in pediatrics has largely been extrapolated from adult studies that rely on imaging criteria, with specific limitations to the imaging techniques coming from interobserver variability in interpretations and the lack of standardized definition of what criteria on imaging would constitute definite CP (5). This highlights the knowledge gaps and the urgent need for CP blood biomarkers in children.

Recent advances in omics technologies, including genomics, proteomics, and metabolomics, have opened new avenues for biomarker discovery. We have previously reported on a panel of chemokines and cytokines in the pediatric AP population, while other authors have employed similar proteomic assays to examine changes in adult patients with CP to identify unique immune protein profiles in patients with CP (6, 7). Similar efforts of utilizing targeted proteomics platforms in children with CP are lacking and can help unravel the pathological changes that occur in the CP disease state.

In this study, we sought to elucidate, through an exploratory analysis, the immunomodulatory proteins associated with inflammatory responses in children with CP that may serve as disease biomarkers. We also aimed to compare immunoproteins in plasma samples from patients with CP versus 2 comparison groups: patients with AP flares and healthy controls (HCs), utilizing a novel NULISAseq platform. By advancing our understanding of the differences between these 3 distinct populations, we endeavored to improve diagnostic capabilities specific to pediatric CP, which may lead to a deeper understanding of the unique changes that occur in this condition and the identification of potential future therapeutic targets.

## Results

A total of 167 samples from 146 patients were included in the analysis, including 71 patients with CP, 55 with AP (of whom follow-up data were available at either 3 or 12 months for 21 patients), and 20 HCs. Demographics for participants in the 3 groups are presented in Table 1. The HC group was younger than either the CP or AP group, and the HC group was noted to have a lower BMI than the AP and CP cohorts, but otherwise there were no statistically significant differences between the groups (Table 1).

**Figure 1:**
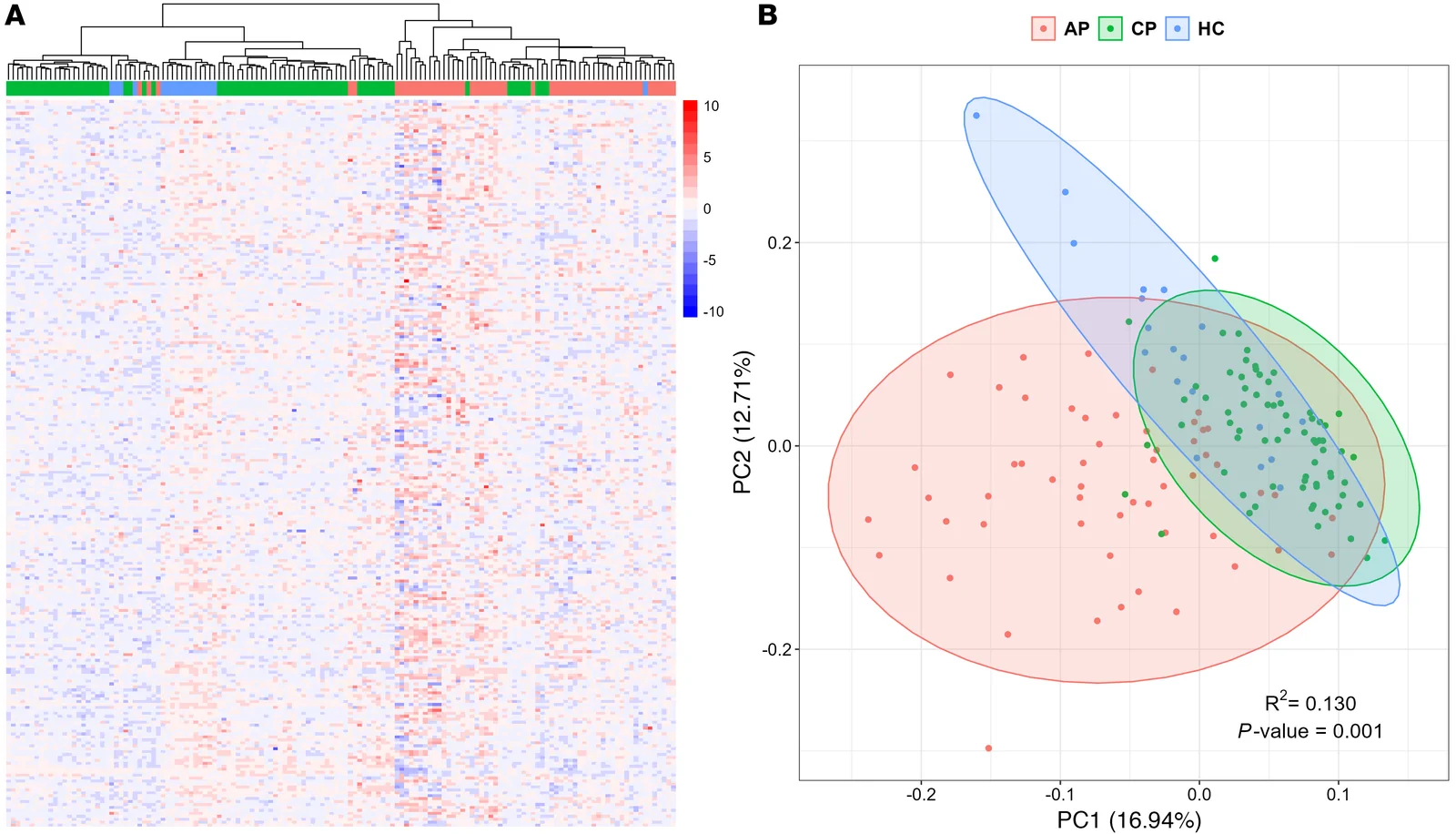
Distinct plasma protein expression profiles differentiate AP, CP, and HC. (**A**) Heatmap of protein expression levels according to cohort membership after adjustment for age and BMI percentile. Rows correspond to log_2_-transformed protein expression levels and columns to samples. Hierarchical clustering of samples was performed using Ward’s method. The column/sample-level annotations show the cohort membership (red = AP, green = CP, blue = HC) and the row/protein-level annotations show the log_2_-transformed NULISA protein quantification expression. The sample-level clustering highlights the overall/multivariate protein expression profiles are more similar within, than between, groups. (**B**) Principal component analysis (PCA) of the first 2 principal components comprising 30% of variation separated by group. Red = AP, green = CP, blue = HC. Overall test: *R*^2^ = 0.130, *P* = 0.001; AP vs. HC: *R*^2^ = 0.073, *P* = 0.001; CP vs. HC: *R*^2^ = 0.073, *P* = 0.001; AP vs. CP: *R*^2^ = 0.110, *P* = 0.001; CP vs. AP + HC: *R*^2^ = 0.087, *P* = 0.001.

The initial NULISAseq analysis was performed for 247 analytes (Supplemental Table 1; supplemental material available online with this article; https://doi.org/10.1172/jci.insight.198911DS1). To test whether the 3 groups had differences in the overall analyte signatures, we performed unsupervised hierarchical clustering analysis and principal component analysis (PCA). There was a clear separation between the 3 groups, AP, CP, and HC, by both methods (Figure 1, A and B). In our PCA data, ordination of the Euclidean distance–based protein-expression profiles for PC1 and PC2 explained 17% and 13% of the variance, respectively, while group membership (AP, CP, and HC) explained 13% of the multivariate distance structure (*R*^2^ = 0.13). Although each axis only accounted for a modest share of the total variability, as is common in complex, high-dimensional biological data, meaningful separation of the groups was observed in the ordination, supporting the interpretation that these first 2 components capture biologically relevant differences in overall protein expression patterns.

**Figure 2:**
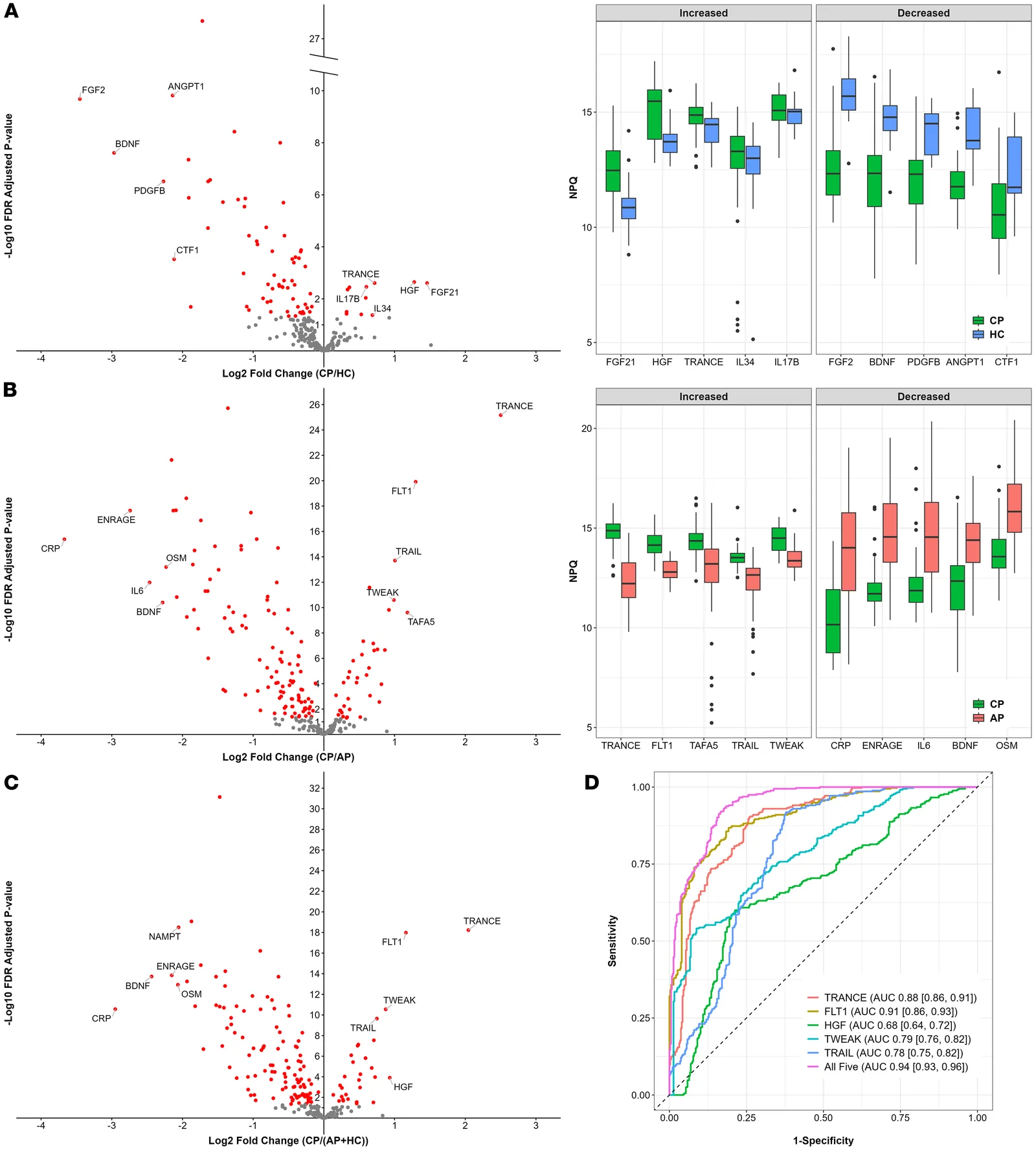
Differentially expressed plasma proteins distinguish CP, AP, and HC. (**A**) Volcano plot for CP (*n* = 71) versus HC (*n* = 20). This highlights proteins with FDR-adjusted *P* < 0.05 in red and labels increased and decreased proteins. Overall differential protein expression with specific box-and-whisker plot among proteins with *q* value less than 0.05, top 5 increased and decreased proteins in CP (green) versus HC (blue). (**B**) Volcano plot for CP versus AP (*n* = 55). This highlights proteins with FDR-adjusted *P* < 0.05 in red and labels increased and decreased proteins. Overall differential protein expression with specific box-and-whisker plot among proteins with *q* value less than 0.05, top 5 increased and decreased proteins in CP (green) versus AP (red). (**C**) Volcano plot for CP versus (AP + HC). This highlights proteins with FDR-adjusted *P* < 0.05 in red and labels increased and decreased proteins. (**D**) ROC curves for top 5 increased proteins in CP compared with AP + HC, showing the 95% CI and AUC for each protein as well as combined: TNF-related activation-induced cytokine (TRANCE), Fms-related receptor tyrosine kinase 1 (FLT1), hepatocyte growth factor (HGF), TNF-related weak inducer of apoptosis (TWEAK), and TNF-related apoptosis-inducing ligand (TRAIL). All box-and-whisker plots display median and interquartile ranges (IQRs).

To test which markers are unique to the CP population, we compared CP to the 2 other cohorts, separately and then combined (AP and HC). The full results for all proteins examined and their pairwise comparisons are presented in Supplemental Table 2. Volcano plots show all proteins, and the most significant proteins are labeled. Figure 2A shows the volcano plot for CP and HC comparisons, with the top 5 increased and decreased proteins. Figure 2B shows the volcano plot for CP and AP comparisons along with the top 5 increased and decreased proteins. A volcano plot of CP versus the 2 groups (HC and AP) combined showed separation for CP signals from the other groups (Figure 2C). Receiver operating characteristic (ROC) curves were created for each of the top 5 proteins: tumor necrosis factor–related (TNF-related) activation-induced cytokine (TRANCE, also known as receptor activator of NF-κΒ ligand [RANKL]), Fms-related receptor tyrosine kinase 1 (FLT1), hepatocyte growth factor (HGF), TNF-related weak inducer of apoptosis (TWEAK), and TNF-related apoptosis-inducing ligand (TRAIL) (Figure 2D). All 5 proteins performed well in differentiating CP, and the 5 proteins combined had an AUC of 0.94 (0.93–0.96) (Figure 2D).

**Figure 3:**
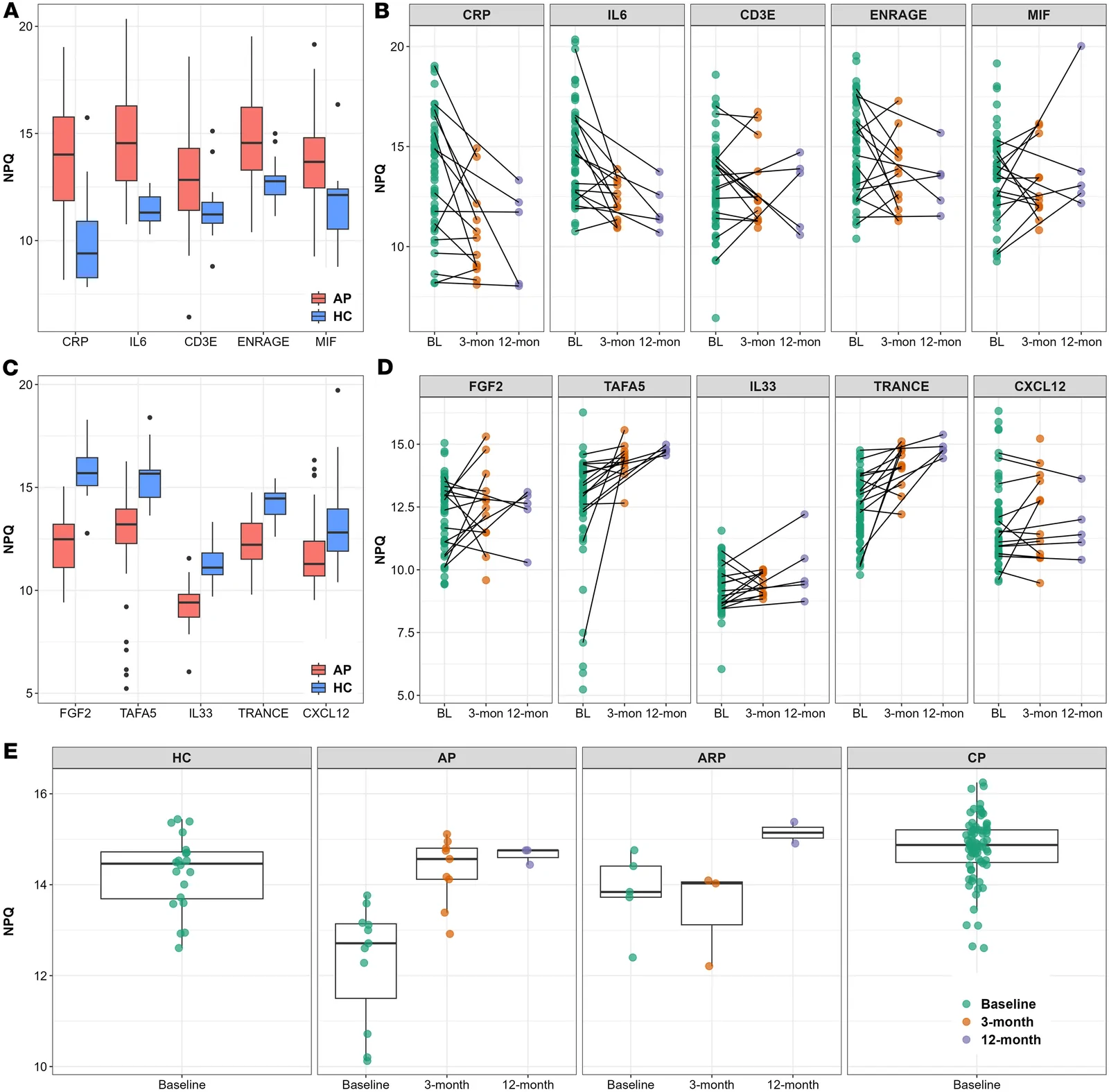
Longitudinal protein alterations following AP reveal disease progression signatures. (**A**) Box-and-whisker plots of the 5 most elevated proteins in AP (*n* = 55, shown in red) and HC (*n* = 20, shown in blue). (**B**) Differential expression of proteins in AP cohort at follow-up visit (either 3 or 12 months). (**C**) Box-and-whisker plots of the 5 most downregulated proteins in AP (red) and HC (blue). (**D**) Differential expression of proteins in AP cohort at follow-up visit (either 3 or 12 months). (**E**) Box-and-whisker plot showing change from baseline for AP cohort over time for TRANCE compared with HC and CP (*n* = 71), separating the patients who developed known ARP (*n* = 5) from the AP cohort in the longitudinal analysis. All box-and-whisker plots display median and interquartile ranges (IQRs).

Next, we analyzed the AP cohort and examined the specific proteins that were most increased and decreased in comparison with the HC group (Figure 3, A and C). Then, within the cohort of patients who presented with AP and had follow-up samples available (after resolution of the AP), we examined the change over time in the significant proteins and plotted these on a spaghetti plot, which showed an increase over time. TRANCE levels, which were significantly decreased in AP versus HCs, increased after resolution of AP (Figure 3, B and D). Among the 21 patients for whom we had follow-up clinic visit data at either 3 or 12 months, it was determined that 5 patients went on to develop acute recurrent pancreatitis (ARP) based on presentation for additional pancreatitis episodes. The levels of TRANCE were then compared between the HCs, the patients who had AP but did not progress to ARP, the patients who did progress to ARP, and the CP group (Figure 3E). There was no statistically significant difference in the follow-up cohort and either the HC or the CP cohort, but there is a trend that emerges where patients with AP will have decreased expression of TRANCE during the acute phase of the disease, which then increases over time and trends towards the levels seen in the HC cohort, continues to rise in the small ARP cohort at 12 months, and then the CP cohort ends up with a higher expression than that seen in either the AP or HC groups (Figure 3E).

**Figure 4:**
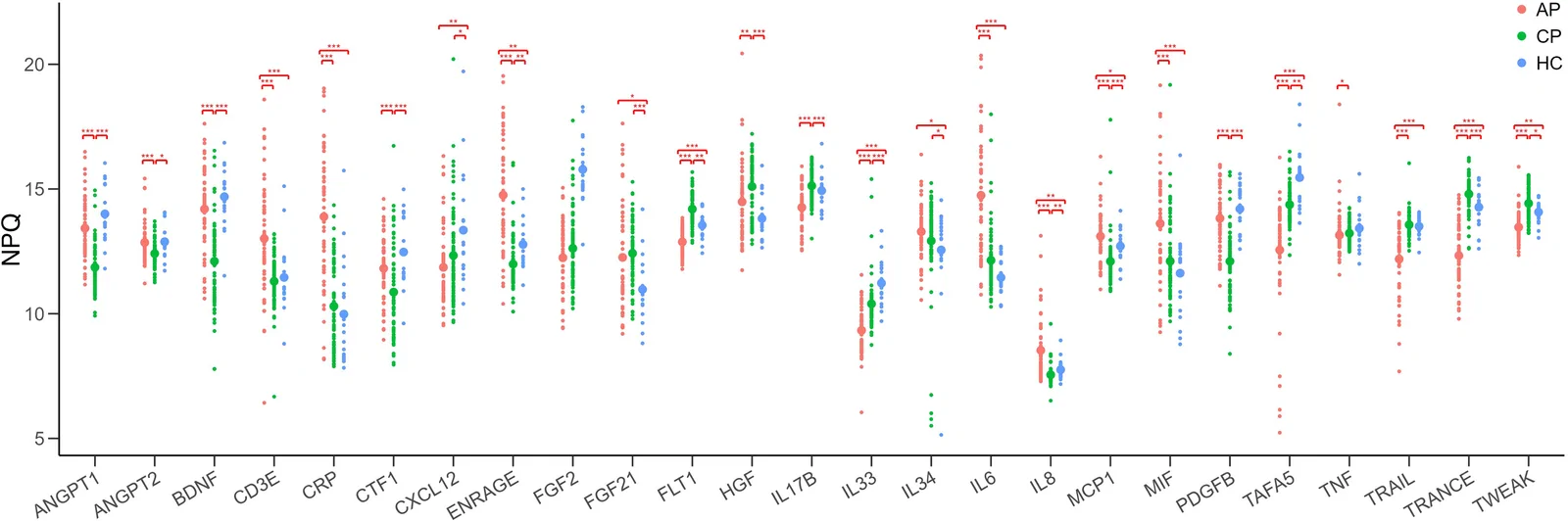
Altered circulating immunomodulatory protein signatures across AP, CP, and HC. Combination of identified increased and decreased immunomodulatory proteins in each of the AP and CP groups compared with the HC group as well as the cytokines previously studied in the MoSAIC trial, showing significant pairwise comparisons. *P < 0.05; **P < 0.01; ***P < 0.001. Benjamini-Hochberg FDR-corrected *P* values were used to account for multiple testing. The plot shows mean and individual observations.

To understand the relationship of these analytes to each other and to prior studies of AP and CP cases (8), we selected significantly increased and decreased markers, particularly the top 5 identified increased and decreased cytokines in each of the AP and CP groups compared with the HC group as well as the cytokines previously identified in the MoSAIC trial: IL-8, HGF, angiopoietin-2 (ANGPT2), IL-6, and MCP-1 (Figure 4) (8). Our comparisons indicate that the findings from our NULISAseq analysis align with the protein markers observed in the MoSAIC trial. Specifically, levels of IL-8, IL-6, and MCP-1 were significantly increased in AP compared with HC. Additionally, these comparisons helped us identify trends in the markers that were significantly increased or decreased in both AP and CP when compared with HCs.

**Figure 5:**
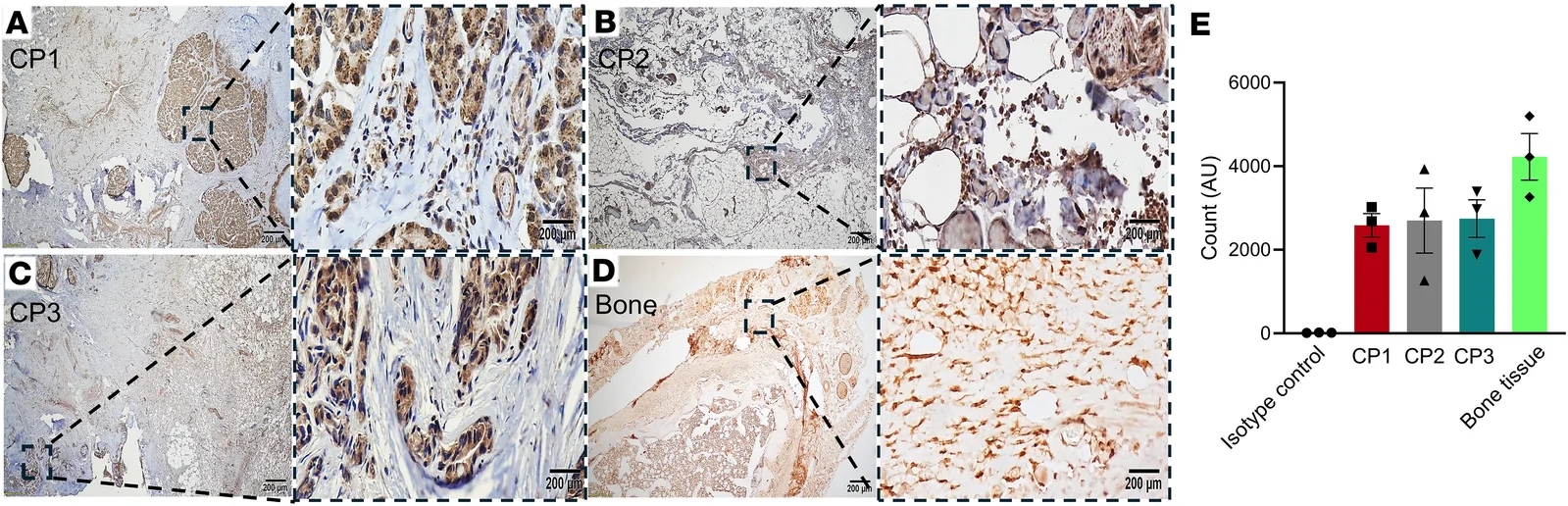
Immunohistochemistry showed expression of TRANCE in pediatric chronic pancreatitis tissue samples. (**A**–**C**) Expression of TRANCE was detected in chronic pancreatitis patients’ tissue samples (3 representative tissues from different patients are shown). (**D**) Normal bone tissue was used as positive control. Scale bars: 200 μm. (**E**) Quantification of TRANCE-positive signal in CP and healthy bone tissue.

Furthermore, we performed immunohistochemistry to detect TRANCE expression in the pancreatic tissue. Figure 5 shows that TRANCE expression was elevated in CP tissues in both fibrotic and nonfibrotic areas from 3 different participant samples in comparison with healthy bone (Figure 5, A–E, n = 3; healthy bone tissue was used as a positive control). TRANCE staining was observed in acinar cells as well as in few infiltrating immune cells. These data suggest that the observed TRANCE in the plasma may arise from multiple sources, and the pancreas could be one of them.

**Figure 6:**
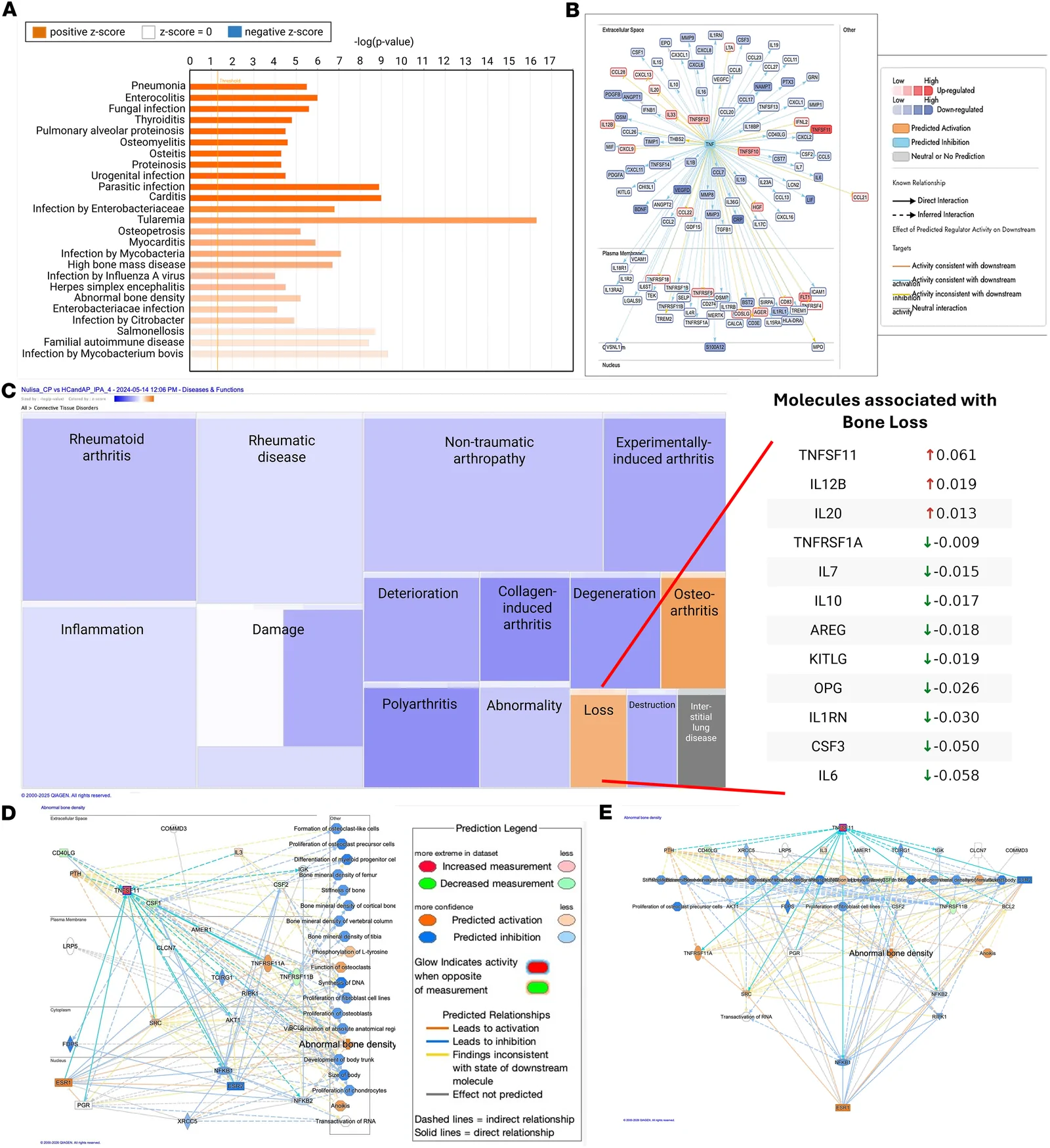
Ingenuity pathway analysis (IPA) highlights disease pathways and bone loss–associated molecules found in CP compared with AP and HC. (**A**) Top positively activated disease pathways in CP versus AP and HC. (**B**) Subcellular layout of an Upstream Regulator network with 4 main compartments. (**C**) Activated disease and functional pathways include “bone loss,” and molecules associated with bone loss are listed. (**D**) Predicted upstream regulator (TRANCE) of the abnormal bone density disease pathway. (**E**) Causal network for the abnormal bone density disease pathway showing interactions among bone loss–associated proteins and predicted downstream biological processes with TRANCE as a key upstream regulator.

To further understand potential pathological roles of differentially regulated immune analytes in CP, we performed pathway enrichment analyses for proteins obtained from CP versus AP and HC combined. Pathway analysis using Ingenuity Pathway Analysis (IPA) revealed that bone- and infection-related pathways had significantly positive *z* scores (Figure 6A). The subcellular layout of the Upstream Regulator network included 4 compartments (extracellular space, plasma membrane, cytoplasm, and nucleus), with significantly increased TRANCE in the extracellular space, including other TNFs (Figure 6B). The analysis also revealed analytes associated with bone loss, including TRANCE, IL-12B, and IL-20, highlighting bone health– and metabolism-relevant features in CP compared with AP and HC (Figure 6C). Some other top canonical pathways that were found in the pathway analysis were pathogen-induced cytokine storm signaling pathway, granulocyte adhesion and diapedesis, and IL-17 signaling pathway. The network analysis of the abnormal bone density pathway identified TRANCE as a central upstream regulator connected to multiple downstream processes involved in bone remodeling, including osteoclast differentiation, osteoblast proliferation, and bone mineral density regulation (Figure 6D). Consistent with these findings, the causal network for abnormal bone density highlighted coordinated interactions among TRANCE and several inflammatory mediators, supporting a potential mechanistic link between CP inflammation and altered bone metabolism in CP (Figure 6E).

## Discussion

We profiled 247 circulating immune-associated proteins in plasma samples from a large pediatric CP cohort, as well as 2 comparison groups (AP and HC), using a novel and highly sensitive immunoassay, NULISAseq. We identified what we believe are unique immune proteins significantly increased in CP compared with AP and HC, including TRANCE, FLT1, HGF, TWEAK, and TRAIL. Pathway analysis revealed multiple enriched immune- and bone metabolism–related disease pathways in CP compared with the other 2 groups.

Immune signals play critical roles in pancreatitis. Circulating immune signals can provide valuable insights into the underlying pathogenesis of the disease and serve as potential diagnostic and prognostic biomarkers. Research on circulating immune signals is particularly limited in pediatric CP, where non-invasive biomarkers are urgently needed. Imaging remains central to CP diagnosis, while functional tests (such as secretin-stimulated pancreatic function testing, fecal elastase-1) complement imaging. However, imaging features tend to be apparent only at later disease stages, and the biochemical tests have varying sensitivity and specificity (9, 10). Additionally, CP presents with variable clinical phenotypes that complicate reliable diagnosis, underscoring a critical need for early diagnostic and prognostic circulating biomarkers. Emerging serum biomarkers (CTGF, IL-17, MMPs, and MIC-1) have shown potential. However, no single marker achieved greater than 90% sensitivity or specificity; false positives can arise from overlap with other pancreatic diseases and non-pancreatic conditions (6, 11–14). Novel and reliable circulating diagnostic biomarkers could reduce unnecessary testing, enable earlier detection, facilitate management, and prioritize clinical trial enrollment, while they may also reveal therapeutically targetable drivers (15).

Notably, our study indicates that the systemic immune responses in CP differ from those in AP and more closely resemble those of HCs, as demonstrated by the PCA plots in Figure 1. This suggests that systemic immune responses may be subdued despite the presence of chronic local inflammation. Our study utilized NULISAseq, which has been demonstrated to be a sensitive immunoassay (11). We also identified what we believe are unique and novel immune markers in CP (top 5 increased proteins), suggesting their potential as a combined model for pediatric CP.

TRANCE (RANKL) is a member of the TNF superfamily (16) that plays critical roles in immune activation and bone resorption (17). TRANCE’s function can be inhibited by osteoprotegerin (OPG); thus, a high TRANCE/OPG ratio indicates increased bone resorption and can be observed in osteoporosis (18–20). Although serum TRANCE levels are increased in patients with pancreatic necrosis and abdominal infection, there are limited reports on the role and source of TRANCE in pancreatitis (21, 22). FLT1 (VEGFR-1) is primarily expressed on vascular endothelial cells and peripheral blood monocytes and involves pancreatic tumor growth and migration (23, 24). VEGFR-1 signaling is essential for bone marrow formation, osteoclast development, and bone repair (25, 26). Soluble serum FLT-1 levels positively correlate with AP severity and predict organ (kidney) failure (27). We also found several proteins known to play critical roles in regulating bone metabolism in the CP cohort, including OSM (decreased, *q* < 0.001), FGF23 (increased, *q* = 0.021), and OPG (decreased, *q* < 0.001). The changes in circulating levels of these bone-related proteins in CP suggest a potential link to bone remodeling within this pediatric cohort. This finding also reinforces our earlier report indicating lower bone mineral density in children with ARP and CP than healthy peers (28). This underscores the potential impact of pediatric CP on bone health and highlights the need for further research in this area.

The top increased proteins in our study are linked to chronic inflammation and fibrosis. Elevated HGF levels, reported in pancreatitis and pancreatic cancer, are highly expressed in pancreatic stellate cells, explaining elevated levels in pediatric CP with fibrotic tissues (29, 30). TWEAK (TNFSF12), mainly expressed in leukocytes (31), regulates angiogenesis and promotes chronic inflammation and fibrosis (32). While serum TWEAK levels showed conflicting results in AP studies (33, 34), recent reports demonstrate that TWEAK/Fn14 signaling promotes CP fibrosis and progression (35). TRAIL, a cytokine that induces apoptosis and activates NF-κB, is expressed in pancreatic stellate cells during early fibrosis, with receptors strongly expressed in CP exocrine epithelium (36).

Our findings align with previous studies on circulating proteins in CP patients as well as in experimental models, including TWEAK, FGF21, and HGF (6, 35, 37).

TRANCE is expressed by various cells, including osteoblasts, stromal cells, and activated T cells (38–40). High levels of expression are found in lymphoid organs, with lower levels detected in peripheral blood and other tissues (40–42). We found that patients with ARP had higher levels of TRANCE than the patients who had AP but did not progress to ARP at 1 year of follow-up. Although there are limited conclusions that can be offered due to the limited subgroup, it is speculated that T cells are involved in circulating TRANCE levels. If circulating T cells are a major source of TRANCE, then levels may be low in AP due to lymphopenia (43–46). These levels can return to normal during recovery and increase as ARP and CP develop. However, our tissue analysis also shows TRANCE expression in CP tissues, suggesting that circulating TRANCE in pancreatitis may be derived from multiple sources, including the pancreas itself.

While our study adds what we believe are novel findings, there are limitations. The fact that all 71 cases of CP originated from a single center is a limitation of this study. Although samples were collected prospectively, we did not have access to longitudinal samples from the same individuals, tracking the progression from the initial AP to CP development (a limitation even for larger consortiums, since it takes years to track 1 participant from AP to CP and this is not yet available for the research community). The HC cohort was limited to 20 participants, which is substantially smaller than the pancreatitis cohorts (AP, *n* = 55; CP, *n* = 71). Also, significant differences in age and BMI were observed between the HC group and both pancreatitis groups, with HCs being younger and having lower BMI values. Furthermore, the CP dataset lacks longitudinal information on bone disease development, including osteopenia outcomes. While we performed statistical adjustments for age and BMI, these baseline differences may complicate data interpretation. In healthy populations, soluble TRANCE levels exhibit age- and sex-dependent variations, with notably higher levels during early adolescence, particularly in males (47–49). Our subanalysis of TRANCE by age groups showed elevated TRANCE levels in CP compared with HCs across age groups (Supplemental Figure 1). To establish TRANCE as a reliable biomarker for pediatric CP, future studies should employ larger, age- and sex-matched cohorts with comprehensive BMI stratification. Additionally, mechanistic investigations using preclinical models across different developmental stages are warranted to elucidate the distinct roles of TRANCE in pediatric versus adult CP pathogenesis and its contribution to disease-associated bone complications.

We also acknowledge limitations in interpreting the function of elevated circulating immune markers. While TRANCE is categorized in bone remodeling pathways, proteins often have pleiotropic effects across different tissues and disease contexts. Although bone complications affect about 60% of adult CP, the pancreas-bone axis relevance in pediatric CP remains less established, with the exception of our results presented here (28, 50, 51). Therefore, elevated TRANCE levels in our pediatric cohort may reflect alternative or additional functions beyond bone metabolism, including immune cell activation or other inflammatory processes within the pancreatic microenvironment. Further mechanistic studies examining TRANCE in pancreatic tissue, immune cell populations, and bone metabolism markers in pediatric CP patients are needed to elucidate the specific biological roles of this pathway in disease pathophysiology.

Although a statistically powered independent validation cohort and longitudinal validation are warranted for further confirmation, combining TRANCE and other markers with imaging features and/or patient characteristics into a composite biomarker could enhance diagnostic accuracy and prognostic utility. Also, 5 increased markers in CP provided better differentiation of CP from other groups than TRANCE alone, suggesting that a multiple-marker panel, potentially including the 5 increased markers reported here, may be needed for robust CP characterization, especially in CP without pain or persistent radiologic changes. These findings underscore the potential value of developing a combined biomarker panel, validated in diverse cohorts and across longitudinal time points.

In conclusion, our study reveals what we believe are unique circulating proteins that significantly differentiate CP from other comparison groups (AP and HC). These proteins include markers relevant to fibrosis (HGF, TWEAK, and TRAIL) and bone metabolism (TRANCE, FLT1, OSM, FGF23, and OPG). Although further validation and functional studies are required, our findings provide insights into a pathogenic connection between CP, fibrosis, and bone metabolism and suggest potential biomarker candidates that can improve our diagnostic capabilities for pediatric CP. Future extensive longitudinal multicenter studies in pediatric CP are necessary to validate our findings and enhance our understanding of the immune signals in this patient population.

## Methods

### Sex as a biologic variable.

Both biologically male and female patients were included in this study, and the findings are expected to be relevant to both sexes.

### Study design.

Study participants were identified and enrolled in 2 prospective observational cohort studies conducted from April 2015 to April 2024. The prospective study (IRB 2016-9510) included participants with CP meeting the criteria set by INSPPIRE definition — any imaging showing presence of CP plus at least one of the following: (a) presence of abdominal pain suggestive of pancreatic origin; (b) presence of exocrine pancreatic insufficiency; and (c) evidence of endocrine pancreatic insufficiency (52). All of the patients in the CP cohort underwent total pancreatectomy with islet auto transplantation (TPIAT) surgery. On the day of their TPIAT, blood and pancreatic tissue samples were obtained from the patients with CP. The AP registry (IRB 2012-4050) enrolls subjects under the age of 21 years meeting the inclusion criteria for AP (52, 53).

Individuals with no underlying organic gastrointestinal diagnosis and specifically no pancreatic disorders were recruited as HCs from the same procedure center for otolaryngological or dental procedures.

### Patient cohort and sample collection.

Blood samples were collected in EDTA-coated tubes (BD, 366643) from patients with CP on the day of surgery; from patients with AP within 48 hours of hospitalization and from post-AP patients at their 3- and 12-month follow-up visits; and from HCs on their date of center visit. For patients in the ARP cohort, this was assessed based on presentation to the hospital with additional AP flares. The 3- and 12-month data points were clinical visits separate from active flares and were conducted after confirmation that the AP flare had subsided. Plasma was extracted from the blood samples within 1–4 hours of collection using high-speed centrifugation, and the resulting clear supernatant was aliquoted and stored at –80°C.

### NULISAseq assay.

The NULISAseq Inflammation panel (Alamar BioSciences) was used as a multiplexed proximity ligation assay at the Human Immune Monitoring Center at Stanford University, which concurrently measures and quantifies 247 inflammation-linked proteins (54). The experimental protocol was implemented using an automated ARGO HT system (Alamar BioSciences), with each 25 μL of sample being processed on a sample plate (as a singlet) alongside multiple control samples, including 3 sample controls, 4 negative controls, and 3 interplate controls (IPCs). The workflow incorporated next-generation Illumina sequencing technology for parallel target analysis. Data management involved dual software platforms: NULISAseq Analysis Software and Alamar Command Center for processing and interpretation. The analytical pipeline included several normalization procedures and log_2_ transformation to optimize the analytical range. IPC-normalized counts were log_2_ transformed to form a more normal distribution, and these log_2_(IPC)-normalized counts are referred to as NULISA Protein Quantification (NPQ) units. Interplate intensity normalization was performed by first calculating the overall and plate-specific median values for each assay. Plate-specific median values were then subtracted from each sample for each assay to scale each assay to have a plate-specific median value of zero, followed by the addition of the overall median to each sample to normalize the intensity across plates.

### Pathway analyses.

To study the pathways involved, pathway analysis was performed using IPA (Qiagen). The differential expression level of each immune marker from NULISAseq data was calculated as mean differences of the adjusted normalized protein expression (NPQ) value in CP compared with AP and HC groups. Estimated mean differences (fold changes) and *P* values adjusted for an FDR at 5% were used as input for the IPA. We used a core analysis module to determine differentially activated disease pathways and generate a graphical summary based on Fisher’s exact test (*P* value cutoff at 0.05).

### Immunohistochemistry analysis.

Immunohistochemical staining was performed to detect TRANCE expression in pancreatic tissues from patients with CP. FFPE CP tissue sections (5 μm) were stained for TRANCE according to a previously reported protocol (55). Briefly, tissue sections were deparaffinized, rehydrated followed by antigen retrieval in 1× Tris-EDTA, pH 9 (BioLegend, 422704) for 30 minutes and then washed with 1× TBS/0.1% Tween 20 (TBST) followed by an endogenous peroxidase block with 3% H_2_O_2_ for 15 minutes. The sections were blocked with 5% goat serum for 1 hour, then incubated with TRANCE primary antibody (Bioss, bs-0747R-TR; 1:500) overnight at 4°C. This was followed by incubation with anti-rabbit secondary antibody for 30 minutes (Vector Laboratories, PK-6101), followed by incubation with DAB (Vector Laboratories, SK-4105), and then counterstained with freshly filtered Mayer’s hematoxylin (Vector Laboratories, H-3401-500). Normal rabbit IgG was used as isotype control (R&D Systems, AB-105-C; 1:500). The tissue sections were imaged using Olympus BX43 microscope. The DAB staining was quantified using ImageJ software (NIH).

### Statistics.

Demographic and clinical characteristics of participants in each cohort were described using medians with interquartile ranges (IQRs) for continuous variables and frequencies with percentages for categorical variables. Kruskal-Wallis rank-sum tests and Pearson’s χ^2^ tests were used to test differences in demographic and clinical characteristics across cohorts. Ordinations of the first 2 PCA axes were used to cluster samples based on the similarity in protein profiles. Permutational analysis of variance (PERMANOVA) was performed using the adonis2 function in the vegan package (version 2.6.4) to partition the sums of squares for each cohort centroid under the default settings and adjusted for age and BMI percentile (56). A heatmap was created using the pheatmap function in pheatmap package (version 1.0.12) to visualize the protein expression levels after adjustment for age and BMI percentile using the residual method. Hierarchical clustering was performed using Ward’s method. Robust linear regression was used to identify differentially expressed proteins between the AP, CP, and HC cohorts. Models included age and BMI percentile as covariates and Benjamini-Hochberg FDR-corrected *P* values to account for multiple testing. Robust linear mixed effects regression was used to identify proteins changing over time in AP samples. Models included time point (e.g., baseline, 3 months, 12 months), age, and BMI percentile as fixed effects, a random subject-specific intercept to account for the repeated measures design, and FDR-corrected *P* values to account for the multiple testing. A multivariable logistic regression model was used to classify samples by cohort (57). Models were fitted to the top 5 increased or decreased proteins, with performance assessed using 10-fold cross-validation. Classification accuracy and the multiclass AUC were used to evaluate performance. All statistical tests were 2-tailed and *P* values of less than 0.05 considered statistically significant. Differentially expressed proteins were identified using an FDR-adjusted *P*-value threshold of less than 0.05. All analyses were conducted using the R software environment for statistical computing and graphics (version 4.4.0; https://cran.r-project.org/bin/windows/base/old/4.4.0/).

### Study approval.

The study was approved by the Cincinnati Children’s Hospital Medical Center Institutional Review Board (protocols IRB 2016-9510 and IRB 2012-4050).

### Data availability.

The overall data, analytic methods, and study materials will not be made generally available, but can be provided through specific requests of the authors. Values for all data points in graphs are reported in the Supporting Data Values file.

## Author contributions

PRF, BL, FA, MEMF: study concept and design; acquisition of data; analysis and interpretation of data; drafting of the manuscript; critical revision of the manuscript for important intellectual content; statistical analysis. AD, NJO, QD, and VG: study concept and design; acquisition of data; analysis and interpretation of data; critical revision of the manuscript for important intellectual content; statistical analysis. JPG, PC, and SZH: study concept and design; acquisition of data; analysis and interpretation of data; critical revision of the manuscript for important intellectual content. MAEH: study concept and design; acquisition of data; analysis and interpretation of data; drafting of the manuscript; critical revision of the manuscript for important intellectual content; statistical analysis; study supervision.

## Conflict of interest

SZH is a consultant for Triveni Bio.

## Funding support

This work is the result of NIH funding, in whole or in part, and is subject to the NIH Public Access Policy. Through acceptance of this federal funding, the NIH has been given a right to make the work publicly available in PubMed Central.

- NIH/National Institute of Diabetes and Digestive and Kidney Diseases (NIDDK) grants R03DK131156 and K23DK118190 (to MAEH).
- NIH/NIDDK grant P30DK116074 (to the Immune Monitoring Core of the Stanford Diabetes Research Center).

## Supplementary Material

Supplemental data

ICMJE disclosure forms

Supporting data values

## Figures and Tables

**Table 1:**
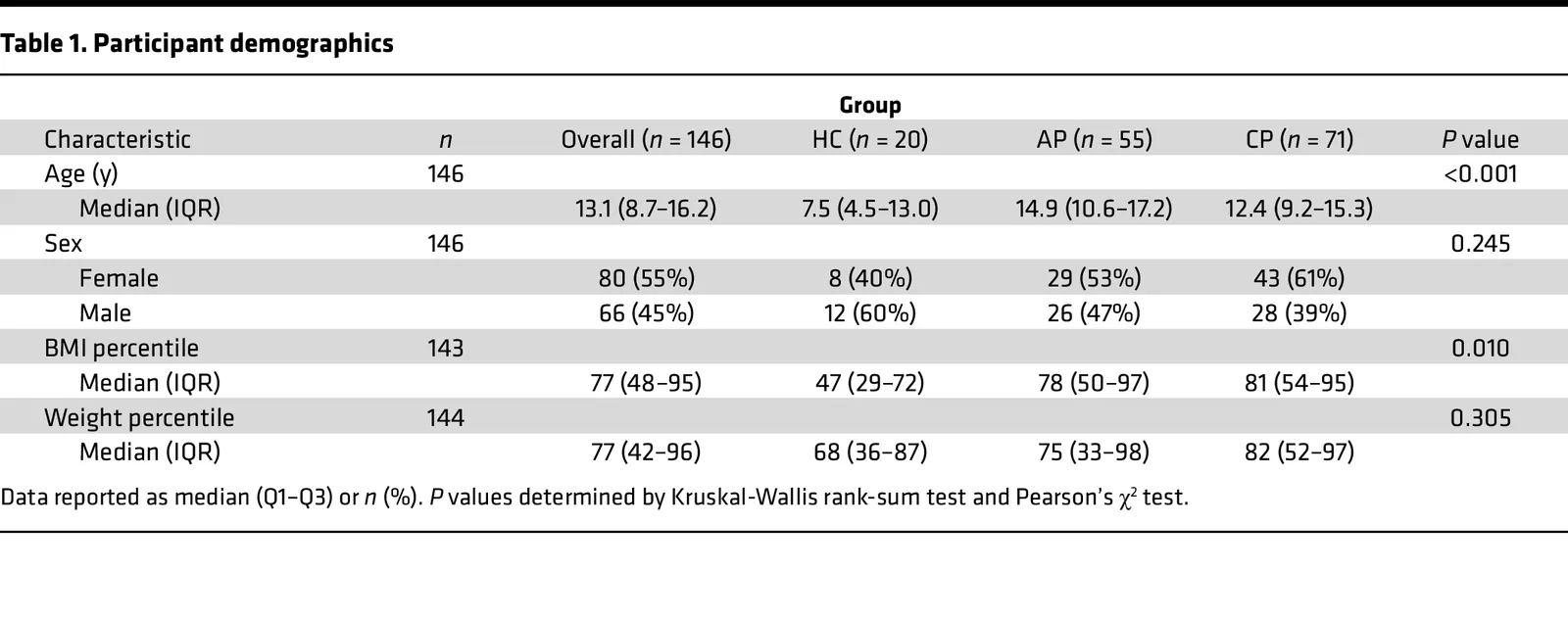
Participant demographics

## Version 1 08/24/2026

Electronic publication
