# Supplementary material for: TRANCE is a unique marker of chronic pancreatitis in children
Source: JCI Insight. 2026 Aug 24;11(16):e198911. doi: 10.1172/jci.insight.198911 (PMC13502190; doi:10.1172/jci.insight.198911)
Supplement: Supplementary file 1 [file jciinsight-11-198911-s219.pdf]

Supplementary Material

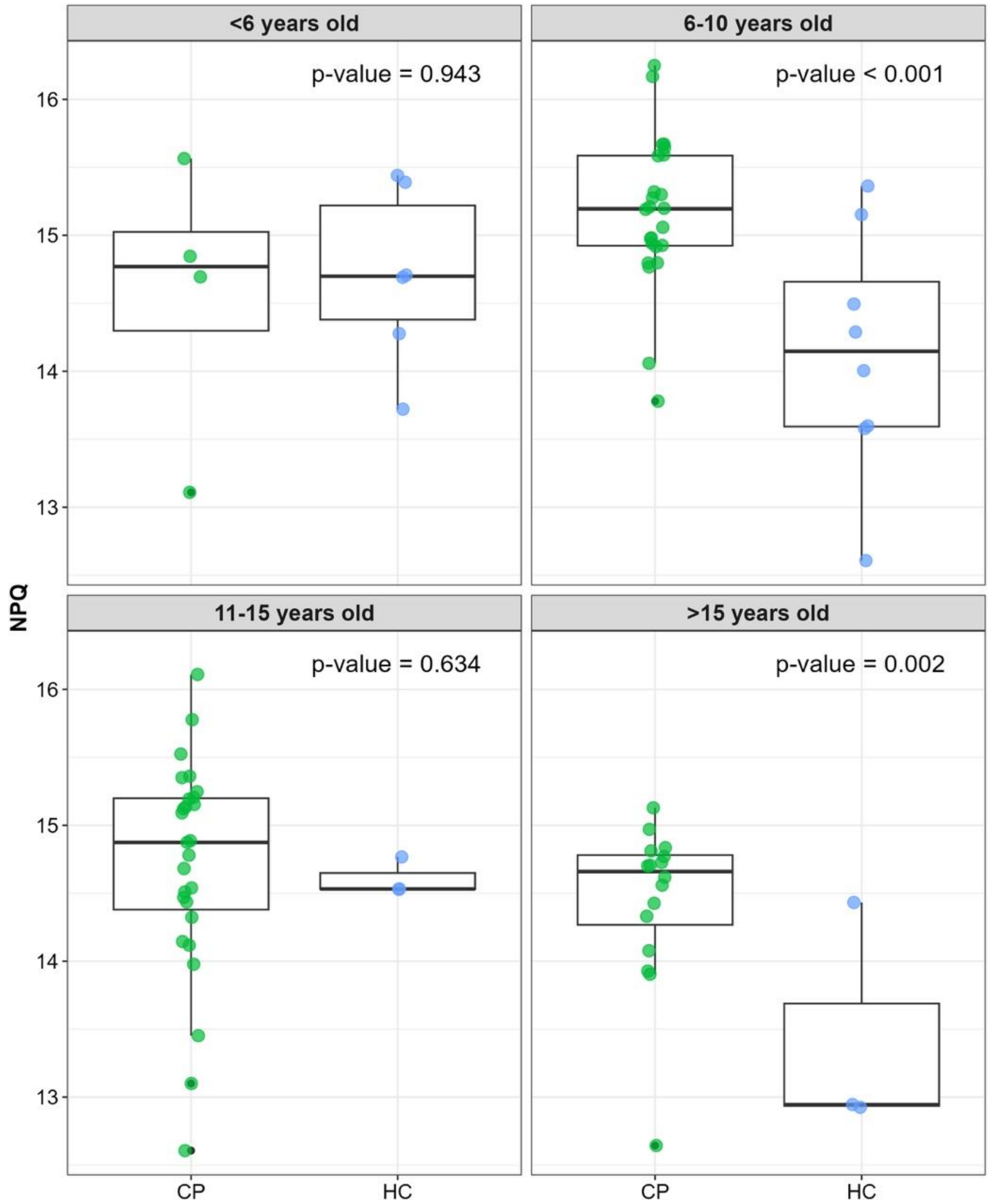

Supplemental Figure 1. Box and Whisker subgroup analysis, separating the CP and HC groups based on age. We found that the CP group had higher levels of TRANCE expression at school age 6-10 and >15, and the sample sizes were too small to find statistical significance in the age less than 6 and ages 11-15 cohorts.

Supplementary material

Supplementary Table 1: List of markers analyzed using NULISAseq Inflammation panel 250 in this study.

S.No.	Name of analytes
1	AGER
2	AGRP
3	ANGPT1
4	ANGPT2
5	ANXA1
6	AREG
7	BDNF
8	BMP7
9	BST2
10	C1QA
11	CALCA
12	CCL1
13	CCL11
14	MCP4
15	CCL14
16	CCL15
17	CCL16
18	CCL17
19	CCL19
20	MCP1
21	CCL20
22	CCL21
23	CCL22
24	CCL23
25	CCL24
26	CCL25
27	CCL26
28	CCL27
29	CCL28
30	CCL3
31	CCL4
32	CCL5
33	MCP3
34	MCP2
35	CD200
36	CD200R1
37	CD27

38	PDL1
39	CD276
40	CD3E
41	CD4
42	CD40
43	CD40LG
44	CD46
45	CD70
46	CD80
47	CD83
48	CD93
49	CEACAM5
50	CHI3L1
51	CLEC4A
52	CNTF
53	CRP
54	CSF1
55	CSF1R
56	CSF2
57	CSF2RB
58	CSF3
59	CSF3R
60	CST7
61	CTF1
62	CTLA4
63	CTSS
64	CX3CL1
65	CXADR
66	CXCL1
67	CXCL10
68	CXCL11
69	CXCL12
70	CXCL13
71	CXCL14
72	CXCL16
73	CXCL2
74	CXCL3
75	CXCL5
76	CXCL6
77	IL8
78	CXCL9
79	EGF

80	EPO
81	FASLG
82	FGF19
83	FGF2
84	FGF21
85	FGF23
86	FLT1
87	FLT3L
88	FLT4
89	FTH1
90	FURIN
91	GDF15
92	GDF2
93	GFAP
94	GRN
95	GZMA
96	GZMB
97	HAVCR1
98	HGF
99	HLA.DRA
100	ICAM1
101	ICOSLG
102	IFNA1..IFNA13
103	IFNA2
104	IFNB1
105	IFNG
106	IFNL1
107	IFNL2..IFNL3
108	IFNW1
109	IKBKG
110	IL10
111	IL10RB
112	IL11
113	IL12B
114	IL12RB1
115	IL12p70
116	IL13
117	IL13RA2
118	IL15
119	IL15RA
120	IL16
121	IL17A

122	IL17A.IL17F
123	IL17B
124	IL17C
125	IL17F
126	IL17RA
127	IL17RB
128	IL18
129	IL18BP
130	IL18R1
131	IL19
132	IL1B
133	IL1R1
134	IL1R2
135	IL1RL1
136	IL1RN
137	IL2
138	IL20
139	IL22
140	IL23
141	IL24
142	IL27
143	IL2RA
144	IL2RB
145	IL32
146	IL33
147	IL34
148	IL36A
149	IL36B
150	IL36G
151	IL3RA
152	IL4
153	IL4R
154	IL5
155	IL5RA
156	IL6
157	IL6R
158	IL6ST
159	IL7
160	IL7R
161	IL9
162	IRAK4
163	KDR

164	KITLG
165	KLRK1
166	KNG1
167	LAG3
168	LAMP3
169	LCN2
170	LGALS9
171	LIF
172	LILRB2
173	TNFB
174	LTA.LTB
175	MERTK
176	MICA
177	MICB
178	MIF
179	MMP1
180	MMP12
181	MMP3
182	MMP8
183	MMP9
184	MPO
185	MUC16
186	NAMPT
187	NCR1
188	NGFB
189	NT3
190	OSM
191	OSMR
192	PDCD1
193	PDCD1LG2
194	PDGFA
195	PDGFB
196	PTX3
197	ENRAGE
198	S100A9
199	SCG2
200	SDC1
201	SELE
202	SELP
203	SIRPA
204	SLAMF1
205	SPP1

206	TAFA5
207	TEK
208	TGFB1
209	THBS2
210	THPO
211	TIMP1
212	TIMP2
213	TLR3
214	TNF
215	TNFRSF11A
216	OPG
217	TNFRSF13B
218	TNFRSF13C
219	TNFRSF14
220	TNFRSF17
221	TNFRSF18
222	TNFRSF1A
223	TNFRSF1B
224	TNFRSF21
225	TNFRSF4
226	TNFRSF8
227	TNFRSF9
228	TRAIL
229	TRANCE
230	TWEAK
231	TNFSF13
232	TNFSF14
233	TNFSF15
234	TNFSF18
235	TNFSF4
236	TNFSF8
237	TNFSF9
238	TREM1
239	TREM2
240	VCAM1
241	VEGFA
242	VEGFC
243	VEGFD
244	VSNL1
245	VSTM1
246	WNT16
247	WNT7A

Supplementary Table 2. Comparison of groups with their coefficient, SE, and q-values for NULISAseq.

	AP vs. CP			CP vs. HC			CP vs. AP+HC		
**Uniprot**	**Coefficient**	**SE**	**q-value**	**Coefficient**	**SE**	**q-value**	**Coefficient**	**SE**	**q-value**
VEGFD	1.36	0.09	<.001	-1.72	0.08	<.001	-1.47	0.09	<.001
TRANCE	-2.50	0.17	<.001	0.72	0.19	0.002	2.04	0.18	<.001
PTX3	2.15	0.16	<.001	-0.94	0.19	<.001	-1.87	0.16	<.001
FLT1	-1.30	0.11	<.001	0.59	0.19	0.009	1.16	0.11	<.001
IL1RL1	1.95	0.17	<.001	-0.17	0.20	0.546	-1.53	0.17	<.001
NAMPT	2.09	0.19	<.001	-1.91	0.27	<.001	-2.06	0.18	<.001
LIF	2.13	0.19	<.001	-0.04	0.14	0.862	-1.52	0.19	<.001
ENRAGE	2.74	0.24	<.001	-0.75	0.24	0.012	-2.15	0.23	<.001
GRN	1.03	0.09	<.001	-0.44	0.14	0.010	-0.90	0.09	<.001
MMP3	1.74	0.16	<.001	-0.17	0.22	0.591	-1.31	0.18	<.001
IL16	0.95	0.09	<.001	-0.26	0.09	0.016	-0.73	0.09	<.001
CRP	3.67	0.37	<.001	-0.67	0.46	0.290	-2.95	0.38	<.001
CSF1	1.17	0.12	<.001	-0.26	0.13	0.126	-0.90	0.12	<.001
CXCL6	1.54	0.16	<.001	-0.95	0.19	<.001	-1.39	0.15	<.001
IL8	0.65	0.06	<.001	-0.17	0.06	0.020	-0.49	0.07	<.001
MCP1	1.17	0.12	<.001	-0.58	0.16	0.004	-1.01	0.12	<.001
TNFSF14	1.83	0.19	<.001	0.03	0.26	0.956	-1.37	0.20	<.001
TRAIL	-1.01	0.11	<.001	0.12	0.11	0.418	0.75	0.10	<.001
MMP8	1.85	0.20	<.001	-0.73	0.21	0.004	-1.47	0.19	<.001
OSM	2.23	0.25	<.001	-1.64	0.31	<.001	-2.07	0.24	<.001
TIMP1	1.49	0.17	<.001	-0.59	0.17	0.003	-1.24	0.16	<.001
ANGPT1	1.61	0.19	<.001	-2.14	0.26	<.001	-1.74	0.18	<.001
IL6	2.47	0.29	<.001	0.34	0.27	0.338	-1.70	0.29	<.001
THPO	0.67	0.08	<.001	-0.57	0.10	<.001	-0.64	0.07	<.001
TNFSF4	-0.65	0.08	<.001	0.06	0.12	0.709	0.49	0.08	<.001
MCP3	1.68	0.20	<.001	-0.79	0.21	0.003	-1.41	0.18	<.001
HAVCR1	1.64	0.20	<.001	-0.26	0.22	0.368	-1.31	0.19	<.001
OPG	0.79	0.10	<.001	-0.45	0.12	0.002	-0.70	0.09	<.001
MMP9	2.08	0.26	<.001	-1.14	0.29	0.001	-1.82	0.23	<.001
OSMR	0.80	0.10	<.001	-0.16	0.10	0.226	-0.62	0.09	<.001
TWEAK	-0.99	0.13	<.001	0.53	0.21	0.040	0.87	0.11	<.001
BDNF	2.28	0.30	<.001	-2.97	0.43	<.001	-2.44	0.27	<.001
PDGFA	1.34	0.18	<.001	-1.61	0.25	<.001	-1.40	0.16	<.001
PDGFB	1.84	0.25	<.001	-2.27	0.36	<.001	-1.94	0.22	<.001
VCAM1	0.50	0.07	<.001	-0.45	0.10	<.001	-0.50	0.06	<.001
IL33	-0.92	0.12	<.001	-0.73	0.16	<.001	0.51	0.14	0.001
IL18BP	0.78	0.11	<.001	-0.27	0.13	0.097	-0.67	0.10	<.001
IL1RN	1.28	0.18	<.001	-0.22	0.22	0.480	-0.99	0.17	<.001
TAFA5	-1.18	0.16	<.001	-0.52	0.19	0.031	0.72	0.17	<.001
BMP7	0.70	0.10	<.001	-1.27	0.16	<.001	-0.80	0.10	<.001
EPO	1.12	0.16	<.001	-0.13	0.20	0.638	-0.85	0.14	<.001
BST2	1.94	0.27	<.001	-0.21	0.21	0.489	-1.44	0.24	<.001
CSF3	1.40	0.20	<.001	-0.86	0.26	0.010	-1.26	0.19	<.001
CST7	1.16	0.17	<.001	-1.11	0.19	<.001	-1.13	0.15	<.001
CXCL1	1.10	0.16	<.001	-0.33	0.22	0.249	-0.88	0.15	<.001
CD3E	1.78	0.26	<.001	0.19	0.25	0.595	-1.07	0.25	<.001
IL15	0.51	0.08	<.001	-0.13	0.08	0.189	-0.40	0.07	<.001
IL18	1.32	0.20	<.001	-0.75	0.28	0.032	-1.15	0.18	<.001
MMP1	1.29	0.20	<.001	-0.41	0.28	0.274	-1.06	0.19	<.001
PDL1	0.45	0.07	<.001	-0.15	0.10	0.258	-0.38	0.06	<.001
TNFRSF9	-0.56	0.09	<.001	0.32	0.12	0.032	0.48	0.08	<.001
TGFB1	0.32	0.05	<.001	-0.26	0.06	0.001	-0.30	0.05	<.001
AGER	-0.70	0.11	<.001	0.22	0.15	0.280	0.59	0.11	<.001
FURIN	0.72	0.12	<.001	-0.37	0.16	0.067	-0.65	0.11	<.001
IL17B	-0.76	0.13	<.001	0.60	0.17	0.003	0.71	0.11	<.001
CXCL9	-0.86	0.15	<.001	0.21	0.20	0.438	0.68	0.14	<.001
IL12B	-0.71	0.12	<.001	0.07	0.15	0.774	0.52	0.12	<.001
THBS2	0.60	0.10	<.001	-0.41	0.11	0.003	-0.58	0.09	<.001
ICOSLG	-0.54	0.09	<.001	0.24	0.11	0.090	0.41	0.08	<.001

KITLG	0.36	0.06	<.001	-0.24	0.08	0.022	-0.33	0.06	<.001
CX3CL1	0.35	0.06	<.001	-0.35	0.08	<.001	-0.36	0.06	<.001
MIF	1.64	0.30	<.001	-0.02	0.43	0.970	-1.23	0.29	<.001
TREM1	0.62	0.11	<.001	-0.13	0.14	0.528	-0.50	0.10	<.001
IL17C	0.91	0.17	<.001	0.44	0.23	0.136	-0.58	0.17	0.002
CCL21	-0.42	0.08	<.001	0.34	0.10	0.004	0.39	0.07	<.001
CCL23	0.59	0.11	<.001	-0.29	0.14	0.091	-0.53	0.10	<.001
AREG	0.48	0.09	<.001	-0.03	0.08	0.774	-0.32	0.08	<.001
CSF3R	0.37	0.07	<.001	-0.27	0.11	0.057	-0.36	0.07	<.001
IL23	0.80	0.16	<.001	-0.29	0.20	0.285	-0.69	0.15	<.001
IFNL2/IFNL3	-0.64	0.13	<.001	-0.10	0.20	0.747	0.50	0.12	<.001
VEGFC	0.33	0.07	<.001	-0.19	0.08	0.067	-0.28	0.06	<.001
MPO	0.67	0.14	<.001	0.24	0.17	0.288	-0.41	0.13	0.005
CD83	-0.36	0.07	<.001	0.17	0.10	0.206	0.29	0.07	<.001
TNFRSF1B	0.45	0.09	<.001	0.02	0.13	0.932	-0.35	0.09	<.001
CXCL13	-0.60	0.13	<.001	-0.32	0.17	0.147	0.34	0.13	0.015
CHI3L1	0.74	0.16	<.001	0.04	0.20	0.898	-0.54	0.15	0.001
TNFRSF18	-0.34	0.07	<.001	-0.01	0.12	0.960	0.26	0.07	0.001
CCL22	-0.47	0.10	<.001	-0.10	0.13	0.599	0.31	0.10	0.005
IL1B	0.47	0.10	<.001	-0.14	0.09	0.280	-0.41	0.09	<.001
CSF2	0.43	0.10	<.001	0.12	0.14	0.532	-0.30	0.09	0.005
LAG3	-0.46	0.11	<.001	-0.19	0.14	0.288	0.27	0.10	0.016
TNFSF18	-0.28	0.06	<.001	0.14	0.08	0.198	0.23	0.06	0.001
CXADR	0.65	0.15	<.001	0.01	0.11	0.956	-0.43	0.12	0.001
IL18R1	0.44	0.10	<.001	-0.16	0.13	0.360	-0.38	0.09	<.001
HLA-DRA	0.68	0.16	<.001	-1.43	0.24	<.001	-0.86	0.15	<.001
GDF15	0.12	0.03	<.001	0.01	0.03	0.847	-0.08	0.02	0.003
CCL28	-0.81	0.19	<.001	-0.32	0.26	0.371	0.46	0.19	0.028
C1QA	0.49	0.11	<.001	-0.56	0.14	0.002	-0.50	0.10	<.001
CALCA	0.58	0.14	<.001	-0.40	0.16	0.046	-0.56	0.12	<.001
IFNB1	0.79	0.19	<.001	-0.68	0.29	0.062	-0.75	0.18	<.001
ICAM1	0.32	0.08	<.001	-0.10	0.11	0.512	-0.29	0.08	0.001
IRAK4	1.42	0.36	<.001	0.04	0.60	0.962	-1.03	0.34	0.006
IL1R2	0.30	0.08	<.001	-0.40	0.09	<.001	-0.33	0.07	<.001
CCL17	0.94	0.24	<.001	-1.06	0.37	0.027	-0.97	0.22	<.001
CXCL2	1.39	0.35	<.001	0.13	0.47	0.862	-0.98	0.32	0.006
GZMB	0.61	0.15	<.001	-1.12	0.20	<.001	-0.75	0.15	<.001
TNFSF13	0.57	0.15	<.001	-1.21	0.20	<.001	-0.72	0.15	<.001
CTF1	1.11	0.30	0.001	-2.12	0.49	<.001	-1.33	0.27	<.001
CCL11	0.36	0.10	0.001	-0.08	0.14	0.678	-0.29	0.09	0.005
TNFB	-0.17	0.05	0.001	0.00	0.07	0.984	0.13	0.04	0.006
CCL25	-0.65	0.18	0.001	-0.61	0.24	0.052	0.34	0.18	0.092
CCL26	0.60	0.16	0.001	-1.06	0.21	<.001	-0.73	0.16	<.001
LCN2	0.35	0.10	0.001	0.00	0.13	0.988	-0.27	0.09	0.008
IL10	0.63	0.18	0.001	0.13	0.19	0.633	-0.44	0.16	0.016
IL20	-0.34	0.10	0.002	0.05	0.14	0.791	0.25	0.09	0.008
TEK	0.22	0.06	0.002	-0.62	0.09	<.001	-0.33	0.06	<.001
CCL20	0.46	0.13	0.002	0.00	0.16	0.996	-0.35	0.12	0.008
TREM2	0.45	0.13	0.002	-0.26	0.17	0.232	-0.41	0.11	0.001
ANGPT2	0.36	0.10	0.002	-0.28	0.14	0.115	-0.36	0.09	<.001
LGALS9	0.27	0.08	0.003	-0.30	0.11	0.032	-0.27	0.07	0.001
IL6ST	0.17	0.05	0.003	-0.15	0.07	0.075	-0.18	0.05	0.001
HGF	-0.78	0.24	0.003	1.28	0.34	0.002	0.93	0.22	<.001
TNFRSF1A	0.32	0.10	0.003	0.00	0.12	0.996	-0.24	0.08	0.008
TNFRSF4	-0.29	0.09	0.006	0.05	0.14	0.843	0.22	0.08	0.022
CEACAM5	0.54	0.18	0.006	-0.34	0.24	0.305	-0.46	0.16	0.010
MCP4	0.45	0.15	0.006	-0.63	0.18	0.003	-0.54	0.14	<.001
MCP2	0.44	0.14	0.006	-0.70	0.18	0.001	-0.54	0.13	<.001
LILRB2	0.20	0.07	0.007	-0.16	0.10	0.220	-0.19	0.06	0.004
CLEC4A	0.26	0.09	0.008	-0.51	0.15	0.004	-0.33	0.09	0.001
SCG2	0.56	0.19	0.008	-1.64	0.26	<.001	-0.83	0.18	<.001

SIRPA	0.27	0.09	0.009	-0.14	0.15	0.506	-0.25	0.08	0.007
IL15RA	0.21	0.07	0.009	-0.10	0.10	0.498	-0.19	0.07	0.012
CXCL16	0.26	0.09	0.011	-0.18	0.13	0.280	-0.26	0.08	0.006
IFNL1	-0.36	0.13	0.011	-0.35	0.20	0.203	0.19	0.12	0.186
IL22	0.90	0.32	0.013	0.53	0.40	0.316	-0.58	0.29	0.081
TNFRSF13B	-0.23	0.08	0.013	-0.22	0.12	0.148	0.10	0.08	0.307
KLRK1	-0.24	0.09	0.013	-0.31	0.12	0.036	0.10	0.08	0.304
IL4R	0.15	0.06	0.013	-0.32	0.07	<.001	-0.21	0.05	<.001
SELP	0.29	0.11	0.015	-0.31	0.18	0.190	-0.31	0.10	0.006
PDCD1	-0.25	0.10	0.019	-0.06	0.14	0.792	0.16	0.09	0.111
IFNW1	0.55	0.21	0.021	-0.20	0.29	0.652	-0.46	0.20	0.043
MERTK	0.21	0.08	0.021	-0.22	0.12	0.165	-0.22	0.07	0.007
IL5RA	0.41	0.16	0.022	-0.11	0.20	0.710	-0.35	0.15	0.035
VSTM1	0.70	0.27	0.022	-0.12	0.44	0.862	-0.58	0.27	0.056
IL2RB	-0.21	0.08	0.028	0.02	0.12	0.932	0.15	0.08	0.116
IL19	0.35	0.15	0.034	-0.39	0.21	0.163	-0.39	0.13	0.008
IL2RA	0.26	0.11	0.036	0.07	0.16	0.776	-0.17	0.09	0.106
FGF23	-0.27	0.12	0.039	0.28	0.17	0.220	0.27	0.11	0.021
IL17RB	0.35	0.15	0.039	-0.34	0.21	0.220	-0.38	0.13	0.011
IL13RA2	0.18	0.08	0.040	-0.19	0.12	0.253	-0.18	0.07	0.028
IL7	0.45	0.19	0.040	-0.43	0.27	0.226	-0.44	0.17	0.021
FGF19	-0.51	0.22	0.043	0.45	0.30	0.264	0.46	0.20	0.038
CCL15	-0.33	0.14	0.043	-0.43	0.18	0.065	0.09	0.14	0.617
WNT16	-0.32	0.15	0.049	-0.06	0.21	0.854	0.23	0.14	0.158
SLAMF1	0.18	0.08	0.052	-0.37	0.13	0.020	-0.24	0.08	0.006
CXCL11	0.69	0.32	0.059	-1.91	0.32	<.001	-1.07	0.31	0.002
CD40LG	0.59	0.28	0.067	-1.09	0.38	0.020	-0.71	0.26	0.012
VSNL1	0.21	0.11	0.088	-0.17	0.09	0.133	-0.22	0.09	0.026
CCL5	0.68	0.41	0.152	-1.88	0.64	0.020	-0.96	0.37	0.019
KNG1	-0.12	0.08	0.191	-0.29	0.11	0.036	0.02	0.08	0.871
IL17RA	-0.09	0.06	0.224	-0.33	0.07	<.001	-0.03	0.06	0.727
FGF21	-0.43	0.31	0.243	1.46	0.39	0.002	0.70	0.29	0.030
CCL16	-0.15	0.11	0.243	0.32	0.12	0.038	0.18	0.09	0.095
NGFB	0.11	0.09	0.255	0.36	0.10	0.004	0.01	0.08	0.913
TNFRSF17	-0.13	0.11	0.290	-0.63	0.17	0.003	-0.03	0.11	0.860
IL36G	0.09	0.08	0.322	-0.56	0.11	<.001	-0.22	0.08	0.016
FLT3L	0.04	0.04	0.413	-0.19	0.06	0.006	-0.07	0.04	0.083
FGF2	-0.22	0.27	0.501	-3.45	0.42	<.001	-0.55	0.31	0.117
CD4	0.04	0.07	0.645	-0.42	0.10	<.001	-0.14	0.07	0.081
TIMP2	-0.03	0.05	0.702	-0.20	0.07	0.032	-0.04	0.05	0.481
IL34	0.08	0.17	0.704	0.69	0.27	0.042	0.09	0.17	0.681
FLT4	0.02	0.08	0.825	-0.34	0.13	0.033	-0.10	0.08	0.266
IL4	0.03	0.17	0.875	-0.50	0.20	0.046	-0.14	0.16	0.453
CCL27	0.02	0.11	0.891	-0.82	0.16	<.001	-0.25	0.11	0.042
IL9	0.00	0.18	1.000	-0.80	0.26	0.013	-0.20	0.18	0.349
